# Response to Comment on “Following Molecular Mobility during Chemical Reactions: No Evidence for Active Propulsion” and “Molecular Diffusivity of Click Reaction Components: The Diffusion Enhancement Question”

**DOI:** 10.1021/jacs.2c02850

**Published:** 2022-08-03

**Authors:** Nasrollah Rezaei-Ghaleh, Jaime Agudo-Canalejo, Christian Griesinger, Ramin Golestanian

**Affiliations:** † Department of NMR-based Structural Biology, Max Planck Institute for Multidisciplinary Sciences, Am Faßberg 11, D-37077 Göttingen, Germany; # Institut für Physikalische Biologie, Heinrich-Heine-Universität Düsseldorf, Universitätsstraße 1, D-40225 Düsseldorf, Germany; ‡ Department of Living Matter Physics, Max Planck Institute for Dynamics and Self-Organization, Am Faßberg 17, D-37077 Göttingen, Germany; ∥ Rudolf Peierls Centre for Theoretical Physics, University of Oxford, Oxford OX1 3PU, United Kingdom

## Abstract

In their Comment (DOI: 10.1021/jacs.2c02965) on two related publications by our group (*J. Am. Chem. Soc.***2022**, *144*, 1380–1388; DOI: 10.1021/jacs.1c11754) and another (*J. Am. Chem. Soc.***2021**, *143*, 20884–20890; DOI: 10.1021/jacs.1c09455), Huang and Granick refer to the diffusion NMR measurements of molecules during a copper-catalyzed azide–alkyne cycloaddition (CuAAC) “click” reaction. Here we respond to their comments and maintain that no measurable diffusion enhancement was observed during the reaction. We expand on the physical arguments presented in our original *JACS* Article regarding the appropriate reference state for the diffusion coefficient and present new data showing that the use of other reference states, as suggested by Huang and Granick, will still support our conclusion that the two reactants and one product of the CuAAC reaction do not exhibit boosted mobility during the reaction.

Reaction-induced boosted mobility is an exciting paradigm that has been comprehensively verified for micrometer-sized objects, while its relevance to the nano- and sub-nanometer scale, i.e., for enzymes and small molecules, is less clear. There have been theoretical studies, e.g., by one of the authors of the present paper,^1,2^ which propose that “molecular swimmers” are possible and lay out the conditions under which they could be experimentally observed. In recent years, a number of experimental studies have reported diffusion enhancement in nanometer objects such as single enzymes.^3−5^ However, these reports have been scrutinized on theoretical and experimental grounds, and, accordingly, the measurability of diffusion enhancement in enzymes in the existing experimental setups has been critiqued,^6−9^ most recently in ref (10). On this background, a recent *Science* paper from Granick’s group reporting pronounced diffusion enhancement for small molecular reactants has drawn the attention of a larger scientific community to the idea of molecular diffusion enhancement.^11^ A particular case in their original and follow-up papers is the Cu(I)-catalyzed azide–alkyne cycloaddition (CuAAC) “click” reaction, for which the components of the reaction are claimed to exhibit diffusion enhancement in NMR diffusion measurements.^11−13^ These reports have been the subject of a series of critical exchanges, in which the existence of measurable diffusion enhancement for the CuAAC reaction components has been seriously debated.^12−18^ Recently, through carefully designed NMR diffusion measurements and analyses, including devising two novel post-acquisition NMR analysis methods, we reported that there was no measurable enhanced diffusion of the two reactants (alkyne and azide) and single product (triazole) of the CuAAC click reaction, and the observed alterations in their diffusion coefficients (*D*_eff_) pointed to the role of relatively large reaction intermediates diffusing more slowly than both the reactants and the product.^19^ In their Comment on our article, Huang and Granick present a number of criticisms,^20^ which we address below.

## 1 The Choice of Reference State for Reactants

**Figure 1:**
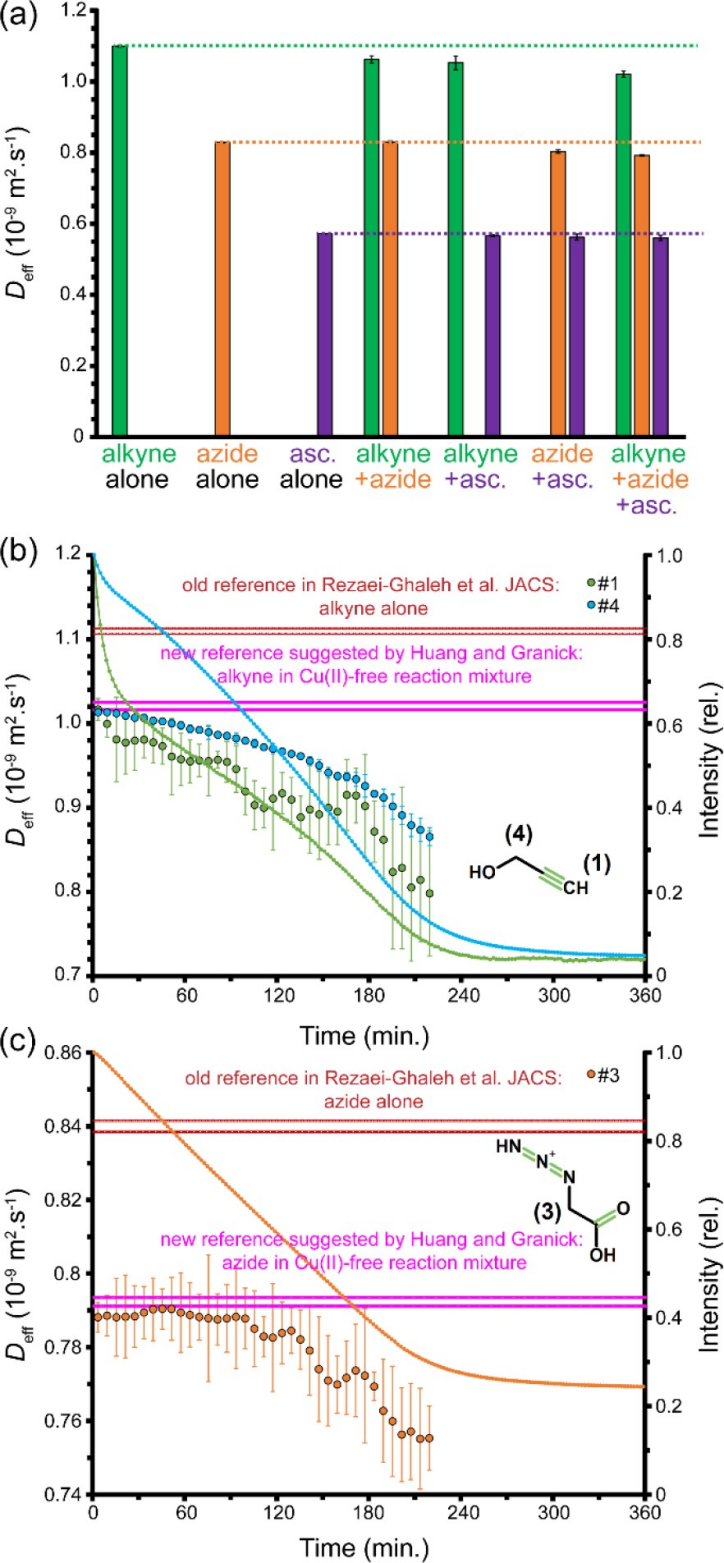
(a) Diffusion coefficient, *D*_eff_, of the reactants (alkyne and azide) and co-catalyst (asc. = ascorbate) measured alone or in the presence of other components. The two reactants show a considerable decrease in their *D*_eff_ values when the other reactant and co-catalyst are present. The *D*_eff_ values obtained in the presence of each reactant alone (“old reference in Rezaei-Ghaleh et al. JACS”) or in the presence of two reactants and co-catalyst but in the absence of catalyst, CuSO_4_ (“new reference suggested by Huang and Granick”) were used as reference states, respectively in our original article^19^ and as suggested by Huang and Granick.^20^ (b, c) Diffusion of alkyne (b) and azide (c), monitored during the click reaction, as shown in Figure 4 of our original article,^19^ but this time including both the old and new reference diffusion coefficients, *D*_0_ (average ± std dev, shown as dashed lines). The *D*_eff_ values of both reactants are smaller than their corresponding new *D*_0_ values over the course of the click reaction. The *D*_eff_ of alkyne shows a decay from the beginning of the reaction, while that of azide remains nearly constant during the first 60–90 min of reaction and then rapidly decreases. The time-dependent changes in signal intensity are shown as lines.

In our article, we used the diffusion coefficient of each reactant in D_2_O in the absence of the second reactant, co-catalyst (sodium ascorbate), and catalyst (copper sulfate) as the reference diffusion coefficient (*D*_0_).^19^ We did not use the limiting diffusion coefficient (*D*_∞_) of reactants toward the end of reaction as *D*_0_, because considering the known mechanism of the click reaction and the formation of various reaction intermediates, we deemed it not to be a physically appropriate reference state, as also previously shown in ref (16). To avoid any further complication arising from the known coordination of alkyne or azide with copper ions or their possible ascorbate-catalyzed redox reactions, we did not use any mixture as the reference state either. Our choice of reference state was criticized by Huang and Granick for being “artificial” and not “physically meaningful”.^20^ The reference state used by us (reactant alone) actually corresponds to the reference state that Wang et al. misleadingly claimed to have used in their original work, in which (on the first page) they defined their measured values Δ*D*_app_/*D*_0_ as “the relative diffusion increase over the Brownian diffusion coefficient of the same molecules”.^11^ Any reader would almost certainly have understood this definition as implying that *D*_0_ corresponds to the diffusion coefficient of the molecule under consideration in isolation. The true meaning of *D*_0_ as the measured diffusion of the corresponding signal at the end of the reaction was only made clear in their subsequent publications.^12,13^ Regarding this, we find it rather disingenuous to use the words “boosted” or “increased” when referring to cases in which the diffusivity is not being compared to a past state but rather to a future state, particularly in cases in which the diffusivity decreases monotonically in time throughout the reaction. Huang and Granick now argue that “the relevant comparison should be the mixture, with and without chemical reaction, because physically this is the more meaningful way to isolate the effects of the chemical reaction.” ^20^ In fact, when we measure diffusion coefficients in different one-, two-, or three-component mixtures (Figure 1a) and use those of the three-component mixture as the reference state as proposed by them, our results are supported even more clearly (Figure 1b,c): the alkyne starts with a *D*_eff_ close to its new *D*_0_ and undergoes a gradual monotonic decay afterward (in particular for the *D*_eff_ associated with its terminal proton, signal #1), and the *D*_eff_ of azide remains close to its *D*_0_ value during the first 60–90 min of the click reaction but shows a rapid decay afterward, in accordance with its later entry point to the reaction cycle or, as suggested in ref (18), due to peak overlap with ascorbate or its oxidation products. Importantly, throughout the course of the click reaction, the *D*_eff_ values of both alkyne and azide remained lower than the new *D*_0_ values, indicating no measurable diffusion enhancement also with respect to the new reference state. As shown in Figure 1a, no other choice among possible reference states would compromise our conclusion that the components of the click reaction do not show any reaction-induced boosted mobility. Indeed, all the changes in their diffusion can be attributed to the role of reaction intermediates. Our results do not reproduce the ∼5% transient diffusivity increase of the azide shown in Figure 2E of ref (13). Moreover, they claim a 50% transient diffusivity increase for 2Cu-alkyne, which is, however, not measured directly but rather indirectly calculated, resulting in quite noisy, potentially unreliable values. In this regard, it is important to note that the variable proton magnetization recovery (due to *T*_1_ relaxation) over the course of reaction, as shown in ref (14), precludes quantitative analysis of NMR signals in terms of reactant and intermediate concentrations. In any case, we believe that not only Fillbrook et al.’s work^18^ and our work,^19^ but also Huang et al.’s own later work,^13^ already debunk a substantial fraction of the results reported in ref (11) for the CuAAC reaction.

## 2 Binding–Unbinding Equilibria

We propose that the measured diffusivity for each signal is consistent with a population-weighted average of the Brownian diffusion of the various components (reactants, reaction intermediates, and product) that carry it, and that changes over time of this measured diffusivity reflect changes in the population distribution of these components as the reaction proceeds. We do not understand why Huang and Granick believe that, according to our proposal, the *D*_eff_ of the reactants “should increase monotonically with time” and that of the product “show slowing down”, or why they think we “do not explain” our proposed mechanism,^20^ because the mechanism is very simple and already explained in ref (19). In short, we observe a monotonic decrease in the diffusivity of the signals corresponding to the reactants, and a monotonic increase in the diffusivity of the signal corresponding to the product. The decrease for the reactants can be explained as being due to an increasing fraction of reactant molecules being in larger, more slowly diffusing complexed forms (Cu-alkyne, 2Cu-alkyne, azide coordinated with 2Cu-alkyne complex) as the reaction proceeds. Conversely, the increase for the product can be explained as resulting from an increasing fraction of product being free, rather than in the form of larger, more slowly diffusing copper triazolide and copper metallacycle complexes, as the reaction proceeds.

**Figure 2:**
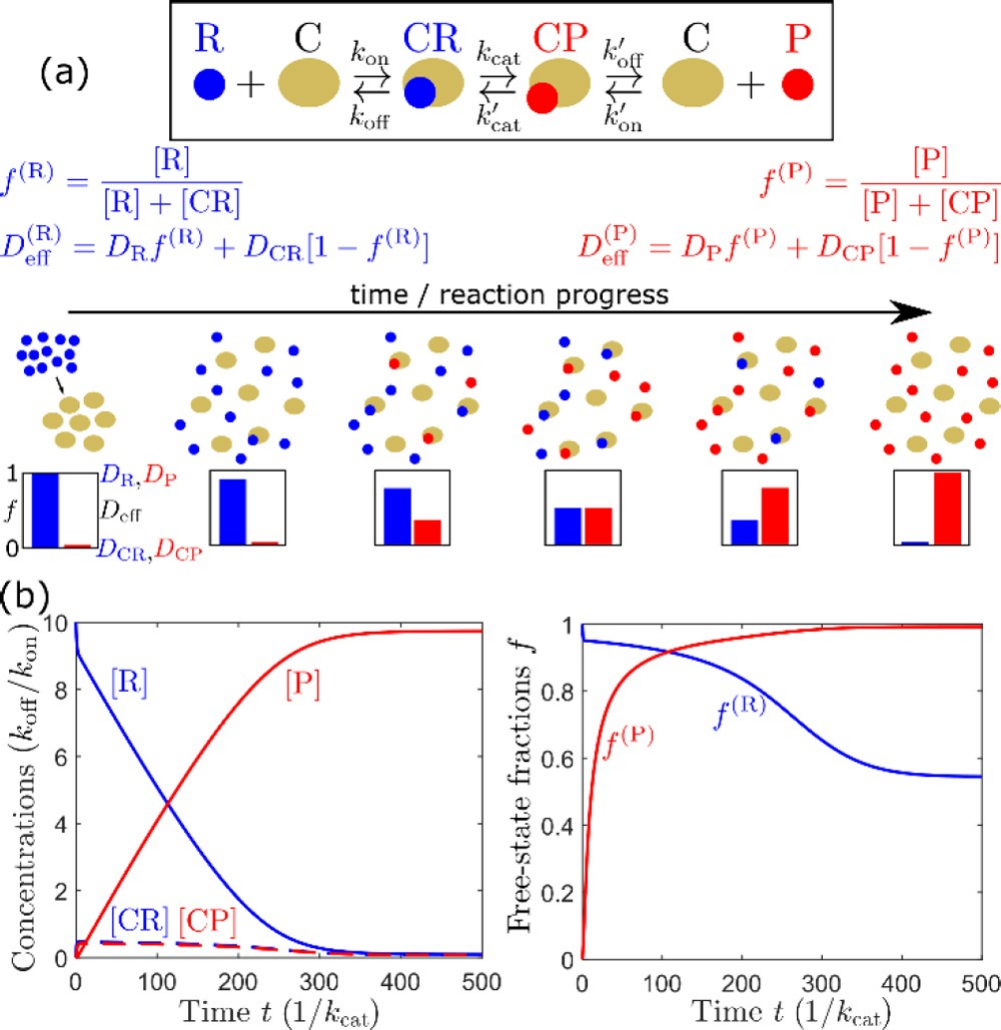
(a) Top: A minimal model for catalytic conversion of a reactant (R) to a product (P) by a catalyst (C) is displayed in the inset, which involves six kinetic rates *k*_*i*_, and five populations: free reactant R, free product P, free catalyst C, reactant–catalyst complex CR, and product–catalyst complex CP. From these populations, the free-state fractions of reactant and product, *f*^(R)^ and *f*^(P)^, can be calculated as shown. The effective diffusion coefficients of each, *D*_eff_^(R)^ and *D*_eff_^(P)^, are under ideal measurement conditions given by a population average of the diffusion coefficients associated with the free state (*D*_R_, *D*_P_) and complex state (*D*_CR_, *D*_CP_), where generally we expect *D*_R_ > *D*_CR_ and *D*_P_ > *D*_CP_. Bottom: Schematic showing how, as the reaction progresses, the free fractions of reactant and product progressively decrease and increase, respectively, causing the respective effective diffusion coefficients to progressively decrease and increase as well. (b) Numerical solution of the kinetic scheme in (a) displaying exactly this behavior. Parameters chosen: *k*_off_ = *k*_off_^′^ = 0.1*k*_cat_, *k*_cat_^′^ = *k*_cat_, *k*_on_ = 100*k*_on_^′^, initial concentrations [C]_0_ = 0.1[R]_0_ = *k*_off_/*k*_on_, [P]_0_ = [CR]_0_ = [CP]_0_ = 0.

While the CuAAC reaction is rather complex and not all the many intervening steps are well described, the basic mechanism we propose can already be understood within a minimal representative model of a catalyzed reaction (Figure 2). Numerical solution of the kinetic scheme shown in Figure 2a, displayed in Figure 2b, shows how, indeed, the fractions of free reactant and product may respectively decrease and increase monotonically as the reaction progresses. Under ideal conditions, in which the free and complex states have the same chemical shift or are in fast exchange, the relaxation rates of both species are the same and constant, and NMR recycle delays are long enough to ensure Boltzmann magnetization recovery (intensity proportional to concentration), these fractions determine the measured effective diffusion coefficient through a population-weighted average. We note, however, that even the simple kinetic scheme in Figure 2 already includes a large number of competing time scales, and thus other behaviors (including non-monotonic ones) of the free-state fractions are possible for different parameter choices. This highlights how even observation of a transient increase in diffusion coefficient is not necessarily a sign of an “active” enhancement and may be explained by complex reaction kinetics. Nevertheless, in all cases, a generic feature of such population averages is that the free fractions of reactant and product may never exceed 1, implying that their effective diffusion coefficients are bounded from above by the diffusion coefficient of their free form.

## 3 Flocculation in Some NMR Experiments

**Figure 3:**
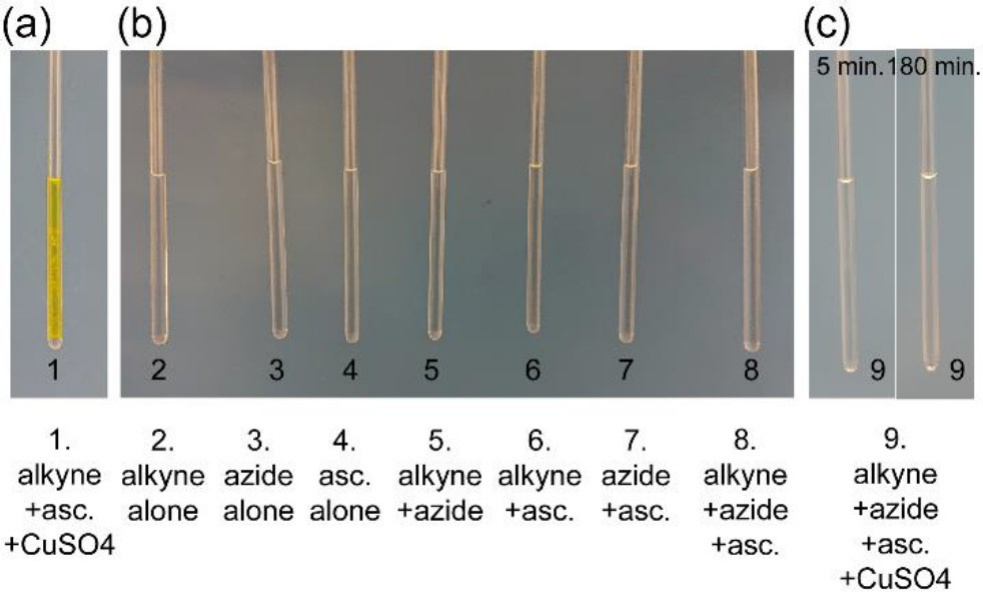
(a) The mixture of alkyne, co-catalyst (asc. = ascorbate) and catalyst (CuSO_4_) but without azide showed flocculation, as correctly pointed out by Huang and Granick.^20^ (b) None of the one-, two-, or three-component samples used for diffusion measurements as reference state in our studies showed flocculation. (c) The four-component reaction mixture (alkyne, azide, ascorbate, CuSO_4_) did not show flocculation during the course of the reaction (e.g., photos taken ∼5 or 180 min after addition of CuSO_4_ are shown).

In another point in their Comment,^20^ Huang and Granick point to the occurrence of flocculation in the mixture of alkyne and catalysts (without azide, see Figure 3a) and rightly state that this system is not suitable for quantitative diffusion measurements. The yellow precipitation is due to the formation of Cu(I) σ-acetylide of propargyl alcohol and its progression toward insoluble, highly colored polymeric compounds.^21,22^ We should, however, highlight the fact that in none of the samples in which we measured diffusion before or during the click reaction was any flocculation present (Figure 3b,c). Therefore, we do not see any relevance for Huang and Granick’s remark in connection with the main results and conclusion of our article.^19^ The purpose of using that sample was only to show whether π-coordination and/or σ-bond formation between alkyne and copper ions without further progression into the click reaction cycle would reproduce the alkyne’s diffusional changes observed during the click reaction. Our results clearly, albeit qualitatively, showed that the mere alkyne–copper binding in the absence of azide was not able to reproduce alkyne’s diffusional changes.^19^

In their Comment,^20^ Huang and Granick could be understood as portraying us as unreasonably resistant to or skeptical of the idea of microscopic energy consumption being transduced into mechanical motion. On the contrary, we have worked for many years in trying to understand the mechanisms by which microscopic objects, from colloids to enzymes, do (or do not) convert chemical activity into motion.^1,2,6,7,23,24^ However, we should still exercise utmost caution and scrutinize the results with physical reasoning, to avoid artificially shoe-horning the idea of active propulsion into molecular-scale systems. For example, the authors of refs (11−13) mention the idea that “boosted motion accompanied by reorientations from rotational Brownian diffusion would produce a random walk with an effective diffusion coefficient larger than that from just Brownian motion”.^13^ The same proposal was put forward by them in order to explain observations of enhanced enzyme diffusion.^25,26^ However, such a mechanism has a very strong dependence on the size of the propelling object. In particular, a particle of size *a* propelling with speed *v* will have an effective diffusion enhancement going as Δ*D* ≈ *v*^2^/*D*_r_, where *D*_r_ ∝ 1/*a*^3^ is the rotational diffusion coefficient of the particle. Dividing this by the Brownian translational diffusion coefficient, *D*_t_ ∝ 1/*a*, one finds that the relative diffusion increase scales with particle size as Δ*D*/*D*_t_ ∝ *v*^2^*a*^4^.^6^ Thus, to obtain the same relative values of diffusion enhancement (say, a few percent), a molecule with *a* = 0.5 nm needs to propel 100 times faster than an enzyme with *a* = 5 nm, or 4 million times faster than an active colloid with *a* = 1 μm. Previous work by us and others^6,8^ has shown how the high speeds required make self-propulsion an unrealistic mechanism for enhanced diffusion already at the scale of enzymes, and this problem only becomes worse at the smaller scale of molecules. This highlights how physical concepts cannot always be transferred across vastly different scales, as appealing as it may be to do so. While we remain open to the idea of chemical activity being transduced into motion at the molecular scale, we believe that every claim must be evaluated and carefully scrutinized according to its own merits. Indeed, the two post-acquisition NMR methods introduced in our Article are intended to enable detecting slight enhancements in molecular diffusion through NMR diffusion measurements less prone to artifacts.^19^ The observations currently existing for the CuAAC reaction are better and more succinctly explained by Brownian diffusion of the various populations that take part in the chemical reaction.
